# Beneficial Effects of Glucagon-Like Peptide-1 (GLP-1) in Diabetes-Induced Retinal Abnormalities: Involvement of Oxidative Stress

**DOI:** 10.3390/antiox9090846

**Published:** 2020-09-10

**Authors:** Hugo Ramos, Patricia Bogdanov, Joel Sampedro, Jordi Huerta, Rafael Simó, Cristina Hernández

**Affiliations:** 1 Diabetes and Metabolism Research Unit, Vall d’Hebron Research Institute (VHIR), 08035 Barcelona, Spain; hugo.ramos@vhir.org (H.R.); patricia.bogdanov@vhir.org (P.B.); joel.sampedro@vhir.org (J.S.); jordi.huerta@vhir.org (J.H.); 2 Centro de Investigación Biomédica en Red de Diabetes y Enfermedades Metabólicas Asociadas (CIBERDEM), Instituto de Salud Carlos III (ICSIII), 28029 Madrid, Spain; 3 Department of Medicine, Universitat Autònoma de Barcelona, 08193 Barcelona, Spain

**Keywords:** diabetic retinopathy, GLP-1, oxidative stress, superoxide dismutase, free radicals

## Abstract

Background: Hyperglycemia-induced oxidative stress plays a key role in diabetic complications, including diabetic retinopathy. The main goal of this study was to assess whether the topical administration (eye drops) of glucagon-like peptide-1 (GLP-1) has any effect on oxidative stress in the retina. Methods: db/db mice were treated with eye drops of GLP-1 or vehicle for three weeks, with db/+ mice being used as control. Studies included the assessment by western blot of the antioxidant defense markers CuZnSOD, MnSOD, glutathione peroxidase and reductase; immunofluorescence measurements of DNA/RNA damage, nitro tyrosine and Ki67 and Babam2 proteins. Results: GLP-1 eye drops protected from oxidative stress by increasing the protein levels of glutathione reductase, glutathione peroxidase and CuZnSOD and MnSOD in diabetic retinas. This was associated with a significant reduction of DNA/RNA damage and the activation of proteins involved in DNA repair in the retina (Babam2) and Ki67 (a biomarker of cell proliferation). Conclusions: GLP-1 modulates the antioxidant defense system in the diabetic retina and has a neuroprotective action favoring DNA repair and neuron cells proliferation.

## 1. Introduction

In recent years emerging evidence has indicated that glucagon-like peptide 1 (GLP-1) exerts beneficial effects in experimental diabetic retinopathy (DR) [1,2,3,4]. The underlying mechanisms involve a downregulation of vascular endothelial growth factor (VEGF), proinflammatory cytokines and proapoptotic signaling, reduction of the excitotoxicity mediated by glutamate and a protective role for the tight junctions and cells of the blood-retinal barrier [1,2,3,4]. However, little is known regarding he effect of GLP-1 on oxidative stress.

Oxidative stress as a result of chronic hyperglycemia play a key role in diabetic complications, including DR [5]. Reactive oxygen species (ROS) and reactive nitrogen species (RNS) are physiologically produced and are needed for redox signaling, but they can also alter the normal cellular homeostasis. For this reason, a precise balance between ROS/RNS production and antioxidant activity is required [6]. The retina is more susceptible to oxidative events than other tissues due to high oxygen uptake and glucose oxidation. In fact, it has been shown that diabetic patients present lower activity of antioxidant enzymes (superoxide dismutase (SOD), glutathione reductase and glutathione peroxidase) and high ROS/RNS levels in the retina [7,8]. Recent experimental evidence suggests that oxidative stress not only contributes to the DR development, but also causes resistance to the beneficial effects of good glycemic control [9].

The aim of this study was to investigate the antioxidant and antinitrosative properties of topical GLP-1 in an experimental model of DR.

## 2. Experimental Section

### 2.1. Experimental Design

A total of 30 diabetic male db/db [BKS.Cg-Dock7^m^ +/+ Lepr^db^/J] mice and 15 non-diabetic mice db/+; [BKS.Cg-Dock7^m^ + Lepr^db^/+] were purchased at the age of 8 weeks (Charles River Laboratories, Calco, Italy). Db/db mice present a mutation in the leptin receptor that triggers obesity-induced type 2 diabetes. The mice had access to ad libitum food (ENVIGO Global Diet Complete Feed for Rodents, Mucedola, Milan, Italy) and filtered water. They were housed at 20 °C temperature and 60% humidity throughout all the study. With the aim of minimizing variability, the animals were randomly distributed (block randomization) in groups of 4 mice per cage. Each cage held absorbent bedding and nesting material (BioFresh Performance Bedding 1/800 pelleted cellulose, Absorption Corp, Ferndale, WA, USA).

### 2.2. Interventional Study

When the mice reached the age of 21 weeks, GLP-1 eye drops (*n* = 15) and vehicle (phosphate-buffered saline (PBS) eye drops (*n* = 15) were randomly dispensed directly onto the superior corneal surface of both eyes with the help of a micropipette. They received one drop in each eye (5 µL) twice daily for 21 days. On the last day of treatment, at the age of 24 weeks, a drop of GLP-1 (2 mg/mL) or vehicle was administered to each eye 1 h before euthanasia. This study obtained the approval of the Animal Care and Use Committee of VHIR (Vall d’Hebron Research Institute, Barcelona, Spain). All experiments were performed in accordance with the guidelines of the European Community (86/609/CEE) and the Association for Research in Vision and Ophthalmology (ARVO).

### 2.3. Retinal Tissue Processing

On the last day of the topical administrations, 8 db/db mice and 4 db/+ were transcardially perfused with paraformaldehyde 4% (Santa Cruz Biotechnology, Dallas, TX, USA), and the eyes were promptly enucleated, fixed again in paraformaldehyde 4% for 5 h and embedded in paraffin blocks. Previously, each animal had received an intraperitoneal injection of 200 µL of anesthesia (a mix containing 1 mL ketamine (GmbH, Hameln, Germany) and 0.3 mL xylazine (Laboratorios Calier S.A., Barcelona, Spain)). The remaining mice (22 db/db and 11 db/+ mice) were euthanized through cervical dislocation, their eyes were instantaneously enucleated, and retinas were separated depending on the experimental purposes. For experiments that required protein samples, retinas were introduced in sterilized PBS pH 7.4 and frozen in nitrogen liquid. For RNA assessments retinas were submerged in TRIzol reagent (Invitrogen^TM^, Carlsbad, CA, USA) and stored at −80 °C until analysis.

### 2.4. Western Blotting

Retinal proteins were extracted through sonication in 80 µL of lysis buffer (phenylmethanesulfonylfluoride (PMSF), 1 mM; NaF, 100 mM; Na_3_VO_4_, 2 mM; all diluted in RIPA buffer (Sigma, St Louis, MO, USA)) and containing 1X protease inhibitor cocktail (Sigma, St Louis, MO, USA). Twenty-five micrograms protein of each sample were loaded in a 10% (*w*/*v*) SDS-PAGE, and electrophoresis was assessed at 90 V and 120 V for 30 and 60 min, respectively. The proteins were then transferred to a polyvinylidene difluoride (PVDF) membrane (Bio-Rad Laboratories, Madrid, Spain) at 400 mA for 90 min at 4 °C and blocked in 5% skimmed milk powder (Central Lechera Asturiana, Siero, Spain) in 0.1% TBS-Tween. Primary antibodies (Table 1) were incubated at 4 °C overnight. Secondary antibodies goat anti-rabbit and goat anti-mouse (Dako Agilent, Santa Clara, CA, USA) were diluted 1:10,000 and the following day they were applied for 1 h at room temperature. Immunoreactive bands were detected using WesternBright ECL kit (WesternBright ECL HRP substrate, K-12045-D50, Advansta, CA, USA). Anti-vinculin (1:7000, sc-73,614; Santa Cruz, Dallas, TX, USA) and anti-cyclophilin A (1:10,000; BML-SA296; Enzo, NY, USA) were used to normalize protein levels. The densitometric analysis was carried out with Image J software (National Institutes of Health, Bethesda, MD, USA).

### 2.5. Immunofluorescence Analysis

Ocular globes were paraffined, sectioned (4 µm) and mounted on poly L-lysine positive charged slides (Leica Biosystems, Nussloch, Germany). The samples were deparaffinized in xylene (VWR, Barcelona, Spain), rehydrated in grade ethanol series (100%, 96%, 70% and 50%), fixed again in ice-cold acid methanol (−20 °C) and washed 3 × 5′ with phosphate-buffered saline 0.01 M (PBS) at pH 7.4. Successively, slides were warmed in a pressure cooker for 4 min at 150 °C in 250 mL of antigen retrieval with sodium citrate 10 mM, pH 6. Then, the sections were blocked with blocking solution (protein block serum-free, X0909 Agilent, Santa Clara, CA, USA) for 1 h at room temperature and they were subsequently incubated overnight at 4 °C with specific primary antibodies (Table 2). Next day, after 3 × 10′ washes in PBS, the samples were incubated for 1 h in darkness with secondary antibodies (Alexa 488 and Alexa 594; 1/600, Molecular Probes, Eugene, OR, USA). The sections were washed again 3 × 5′ with PBS, counterstained with Hoechst 33,342 (bisbenzimide) (Thermo Fisher Scientific, Eugene, OR, USA) and mounted with mounting medium fluorescence (Prolong, Invitrogen, Thermo Fisher Scientific, Eugene, OR, USA) and coverslips. Images were acquired using laser confocal microscopy (Fluoview FV 1000 laser scanning confocal microscope Olympus, Hamburg, Germany) at a resolution of 1024 × 1024 pixels. Immunofluorescence was quantified with Image J software.

### 2.6. Statistical Analysis

Data are presented as mean ± SEM. Quantitative comparisons were analyzed by using Student’s *t*-test and one-way ANOVA followed by Bonferroni’s multiple comparison post hoc test. Statistical significance was set at *p* < 0.05 (*).

## 3. Results

### 3.1. Topical Administration of GLP-1 has no Effect on Body Weight and Systemic Blood Glucose Levels

**Figure 1:**
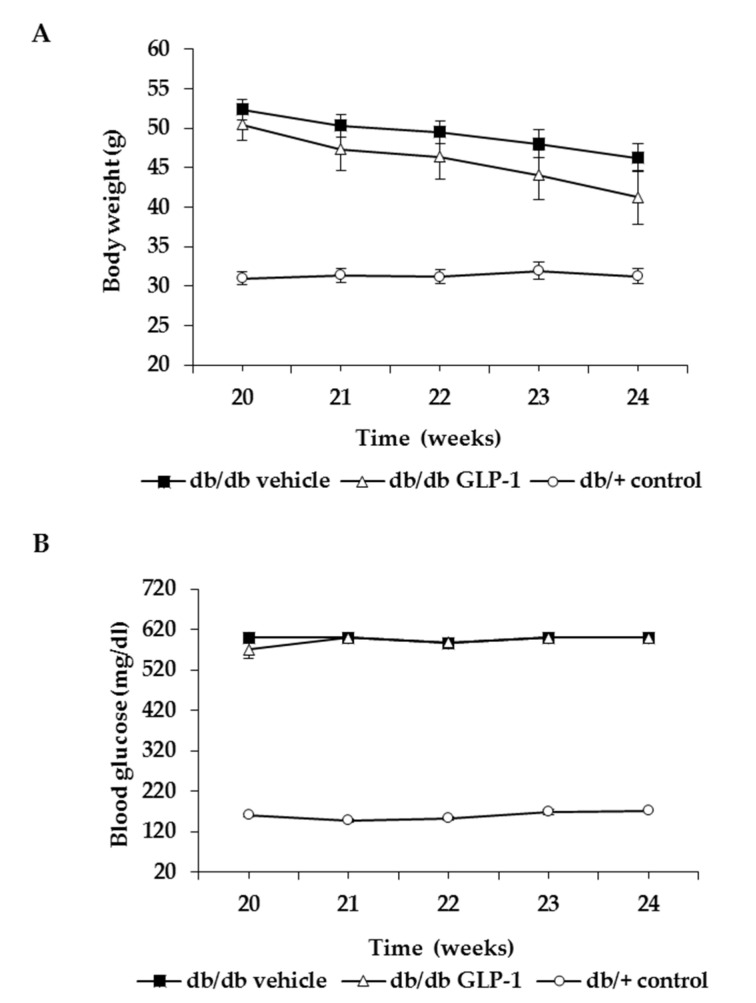
Evolution of (**A**) body weight and (**B**) blood glucose levels in the experimental groups.

No significant difference was observed in body weight and blood glucose concentrations during the experiment between db/db mice treated with GLP-1 eye drops and db/db mice treated with vehicle (Figure 1A,B).

### 3.2. Topical Administration of GLP-1 Reduces DNA/RNA Damage through the Decrease of Reactive Oxygen Species (ROS) and Reactive Nitrogen Species (RNS) Induced by Diabetes in the Retina

**Figure 2:**
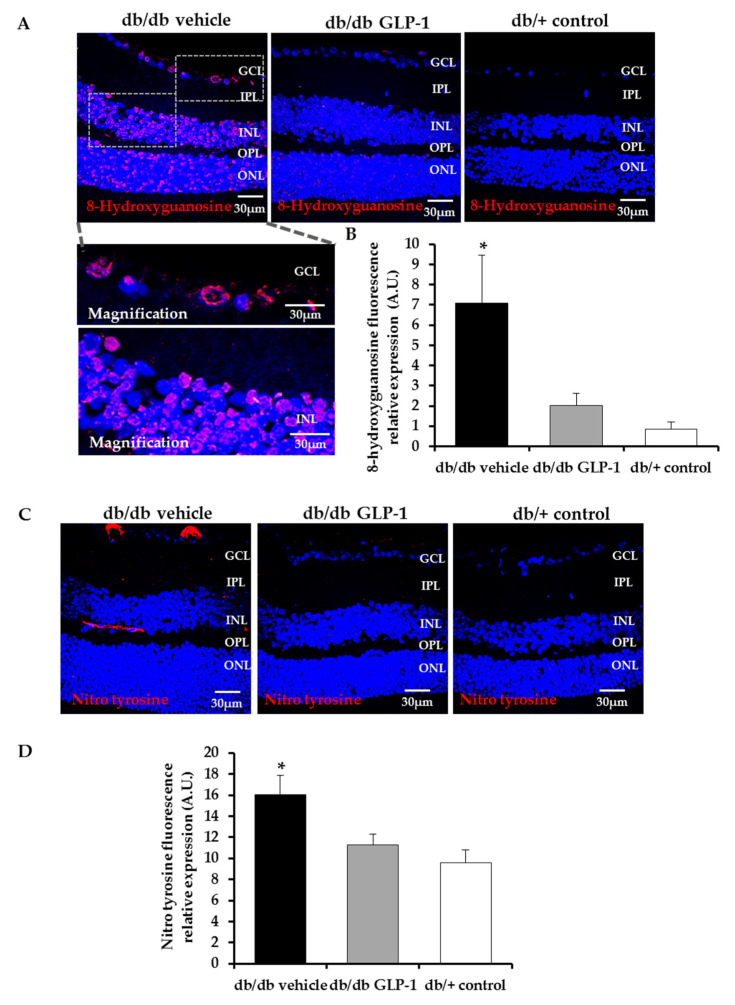
Immunofluorescence analysis of DNA/RNA damage (8-hydroxiguanosine) and nitro tyrosine. (**A**,**B**) Comparison and quantification of 8-hydroxiguanosine (red) protein levels through immunofluorescence among representative samples of diabetic retinas treated with vehicle eye drops (black bars) or GLP-1 eye drops (gray bars) and non-diabetic retinas (white bars). Hoechst staining (blue) was used for nuclei labeling. Optical magnifications of the ganglion cell layer (GCL) and the inner nuclear layer (INL) are also displayed. Scale bars, 30 µm. *n* = 4; (**C**,**D**) comparison and quantification of nitro tyrosine (red) protein levels through immunofluorescence among representative samples of diabetic retinas treated with vehicle eye drops (black bars) or GLP-1 eye drops (gray bars) and non-diabetic retinas (white bars). Hoechst staining (blue) used for nuclei labeling. Scale bars, 30 µm. *n* = 4; * *p* < 0.05. GCL—ganglion cell layer; INL—inner nuclear layer; IPL—inner plexiform layer; ONL—outer nuclear layer; OPL—outer plexiform layer.

The impaired equilibrium between ROS and the antioxidant defenses promotes oxidative stress that affects the structure of several molecules, including nucleic acids. The hydroxyl radicals can damage DNA by converting deoxyguanosine into 8-hydroxyguanosine. Here we provide evidence that this phenomenon occurred in an experimental model of DR (db/db mice) and that the topical administration of GLP-1 could prevented this process (Figure 2A,B).

RNS act similar to ROS in terms of cell damage. In fact, nitro tyrosine protein levels were also increased in the retinas of diabetic mice in comparison with non-diabetic mice. GLP-1 significantly reduced them too (Figure 2C,D).

### 3.3. GLP-1 Eyedrops Protect from Oxidative Stress by Increasing the Protein Levels of Glutathione Reductase, Glutathione Peroxidase and Copper–Zinc and Manganese Superoxide Dismutases (CuZnSOD and MnSOD) in Diabetic Retinas

**Figure 3:**
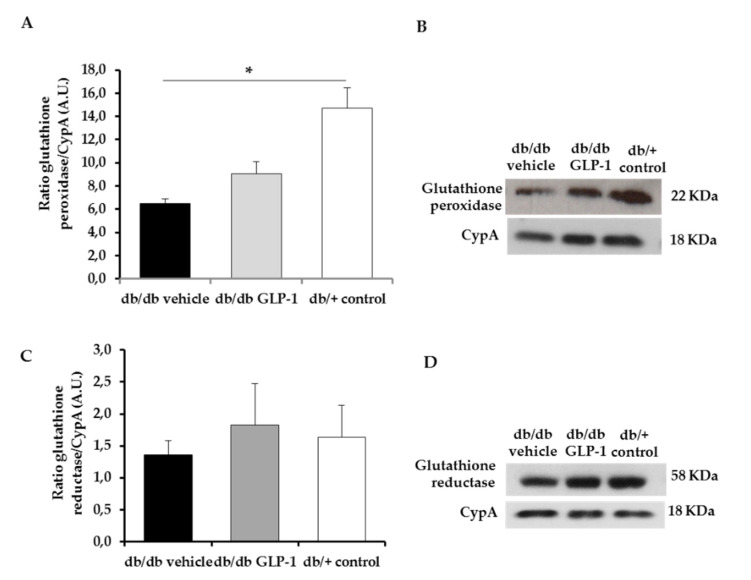
Protein levels of glutathione peroxidase and glutathione reductase. (**A**,**B**) Densitometric analysis and western blot bands of glutathione peroxidase corresponding to retinas of db/db mice treated with vehicle eye drops (black bars), GLP-1 eye drops (gray bars) and to non-diabetic mice retinas (white bars). Protein levels normalized with cyclophilin A. *n* = 3; (**C**,**D**) densitometric analysis and western blot bands of glutathione reductase corresponding to retinas of db/db mice treated with vehicle eye drops (black bars), GLP-1 eye drops (gray bars) and to non-diabetic mice retinas (white bars). Protein levels normalized with cyclophilin A. *n* = 3; * *p* < 0.05.

Glutathione (GSH) effectively scavenges free radicals and other ROS and RNS (e.g., hydroxyl radical, lipid peroxyl radical, superoxide anion and hydrogen peroxide) directly and indirectly through enzymatic reactions. The reduced GSH can be regenerated from oxidized GSH by glutathione redox cycle. However, in the diabetic retina, the enzymes responsible for glutathione redox cycle (glutathione peroxidase and glutathione reductase) are compromised [6,8,10]. We observed a statistically insignificant increase of protein levels of glutathione peroxidase (Figure 3A,B) and glutathione reductase (Figure 3C,D) in diabetic retinas treated with GLP-1 eye drops in comparison with those treated with vehicle.

**Figure 4:**
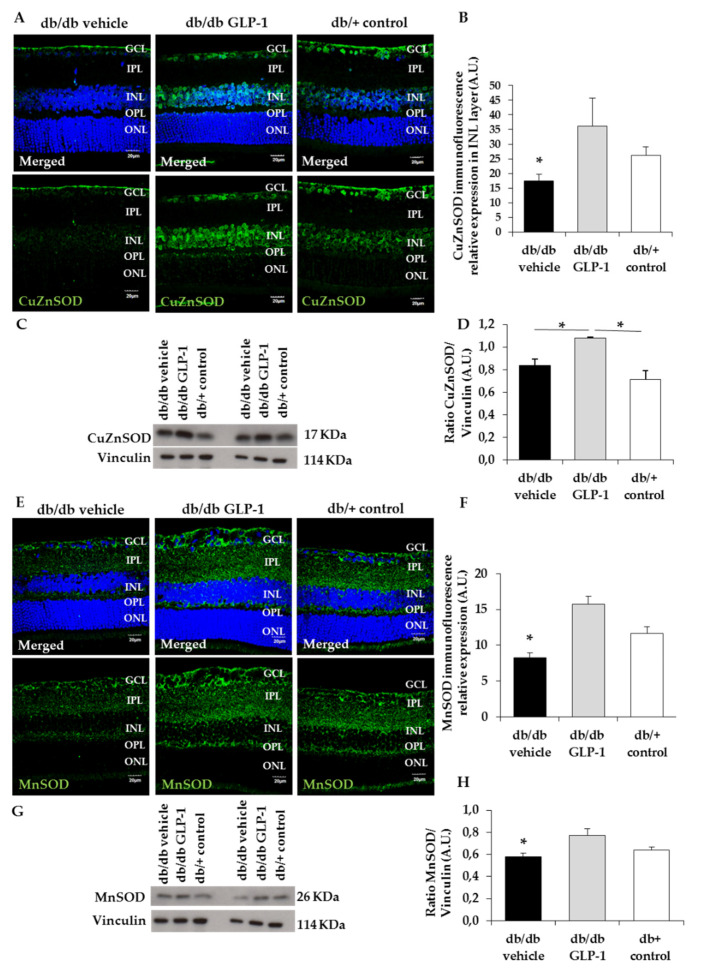
Protein levels of copper–zinc and manganese superoxide dismutase (CuZnSOD and MnSOD) (**A**,**B**) Comparison and quantification of CuZnSOD (green) protein levels through immunofluorescence among representative samples of diabetic retinas treated with vehicle eye drops (black bars) or GLP-1 eye drops (gray bars) and non-diabetic retinas (white bars). Hoechst staining (blue) used for nuclei labeling. GCL—ganglion cell layer; INL—inner nuclear layer; IPL—inner plexiform layer; ONL—outer nuclear layer; OPL—outer plexiform layer. Scale bars, 20 µm. *n* = 4; (**C**,**D**) densitometric analysis and western blot bands of CuZnSOD corresponding to retinas of db/db mice treated with vehicle eye drops (black bars), GLP-1 eye drops (gray bars) and to non-diabetic mice retinas (white bars). Protein levels normalized with vinculin. *n* = 3; (**E**,**F**) comparison and quantification of MnSOD (green) protein levels through immunofluorescence among representative samples of diabetic retinas treated with vehicle eye drops (black bars) or GLP-1 eye drops (gray bars) and non-diabetic retinas (white bars). Hoechst staining (blue) used for nuclei labeling. Scale bars, 20 µm. *n* = 4; (**G**,**H**) densitometric analysis and western blot bands of MnSOD corresponding to retinas of db/db mice treated with vehicle eye drops (black bars), GLP-1 eye drops (gray bars) and to non-diabetic mice retinas (white bars). Protein levels normalized with vinculin. *n* = 3; * *p* < 0.05. GCL—ganglion cell layer; INL—inner nuclear layer; IPL—inner plexiform layer; ONL—outer nuclear layer; OPL—outer plexiform layer.

Ultimately, the radical chain reactions will be blocked by the antioxidant enzymes superoxide dismutase (SOD). The activities of both CuZnSOD, located in the cytosol, and MnSOD in the mitochondria are decreased in diabetic retina [6,8,10]. In the present study, we found that CuZnSOD and MnSOD levels were significantly increased by the topical administration of GLP-1 (Figure 4A–D and Figure 4E–H, respectively); (*p* < 0.05).

### 3.4. Topical Administration of GLP-1 Activates the Expression in the Retina of Proteins Involved in DNA Repair (Babam2) and Cell Proliferation (Ki67)

**Figure 5:**
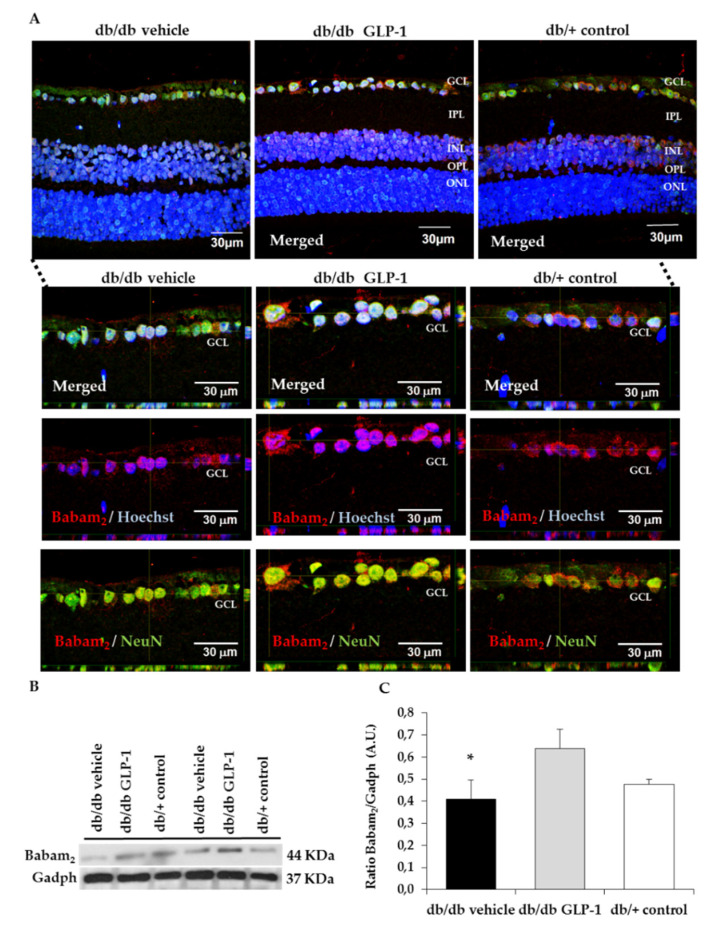
Babam2 protein levels. (**A**) Comparison of colabelling immunofluorescence assay for Babam2 (red) with NeuN (neuronal specific marker) (green) in db/db mice among representative samples of diabetic retinas treated with vehicle eye drops or GLP-1 eye drops and non-diabetic retinas. Nuclei labeled with Hoechst stain nuclei specific marker) (blue). GCL—ganglion cell layer; INL—inner nuclear layer; IPL—inner plexiform layer; ONL—outer nuclear layer; OPL—outer plexiform layer. An orthogonal view of Babam2 to analyze nuclear translocation in GCL of db/db mice treated with vehicle, db/bb mice treated with GLP-1 eye drops and non-diabetic mice are also displayed in this figure. Scale bars, 30 µm. *n* = 4; (**B**,**C**) densitometric analysis and western blot bands of babam_2_ corresponding to retinas of db/db mice treated with vehicle eye drops (black bars), GLP-1 eye drops (gray bars) and to non-diabetic mice retinas (white bars). Protein levels normalized with Gadph. *n* = 3; * *p* < 0.05. GCL—ganglion cell layer; INL—inner nuclear layer; IPL—inner plexiform layer; ONL—outer nuclear layer; OPL—outer plexiform layer.

**Figure 6:**
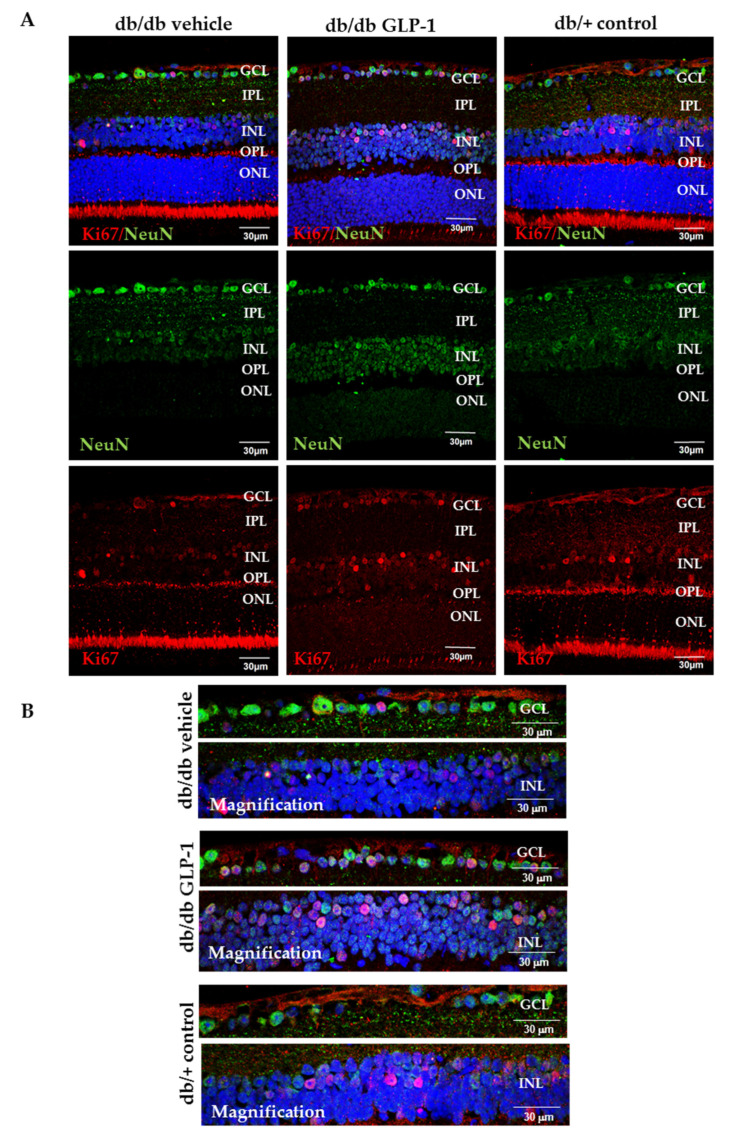
(**A**) Comparison of ki67 (red) protein levels through immunofluorescence among representative samples of diabetic retinas treated with vehicle eye drops or GLP-1 eye drops and non-diabetic retinas. Ki67 is colabelled with NeuN (neuronal specific marker) (green) and Hoechst staining (nuclei specific marker) (blue); (**B**) Optical magnifications of GCL and INL are also presented in this figure. Scale bars, 30 µm. *n* = 4. GCL—ganglion cell layer; INL—inner nuclear layer; IPL—inner plexiform layer; ONL—outer nuclear layer; OPL—outer plexiform layer.

Reactive oxygen and nitrogen species damage cellular macromolecules including DNA. Babam2 or BRE (brain and reproductive organ-expressed protein) is part of the BRCA1, a complex which is implicated in both DNA repair and maintenance of G2/M arrest in reaction to DNA damage [11,12]. For this reason, we wanted to assess Babam2 in our experiment. We found that the retina of untreated diabetic mice had considerably increased DNA/RNA damage compared with controls and that treatment with GLP-1 eye drops significantly increased the protein levels of Babam2 in retina in diabetic mice (Figure 5A–C). Moreover, GLP-1 increases Ki67 protein levels in neuroretina and favors its translocation to the nucleus, thus indicating the promotion of neurogenesis in the diabetic retina (Figure 6A,B).

## 4. Discussion

DR physiopathology embraces several metabolic pathways triggered by the hyperglycemic state. One of them is the overproduction of free radicals and the consequent nitro-oxidative imbalance that leads to cell damage [5]. GLP-1 and GLP-1R agonists have emerged as potential drugs against DR due to their neuroprotective properties, but their effect on DR oxidative stress has not been assessed [1,3,13]. Herein, we provide evidence that an antioxidant effect can be added to the underlying mechanism by which GLP-1R agonists exert their beneficial action on the diabetic retina.

In the present study, we found diabetes-induced oxidative stress resulting in DNA/RNA damage through guanine oxidation and protein damage by tyrosine nitration. In this regard, retinas of diabetic mice presented higher levels of both markers (8-hydroxy-2′-deoxyguanosine and nitrotyrosine, respectively) in comparison with non-diabetic mice, which implies the appearance of nitro-oxidative stress as a consequence of the DR state. Notably, these deleterious effects were prevented by the topical administration of GLP-1. Several studies have reported the relationship between GLP-1 and the decrease of cellular ROS levels [14]. Wang et al. showed that GLP-1 could reduce high-glucose-induced ROS in cardiac microvascular endothelial cells and Erdogdu et al. obtained similar results with exendin-4 (GLP-1 receptor agonist) using human coronary artery endothelial cells [15,16]. Here we demonstrate that the topical administration of GLP-1 prevents the RNA/DNA and proteins damage induced by RNS and ROS in an experimental model of DR. To the best of our knowledge this is the first study showing these beneficial effect of GLP-1 in the diabetic retina. It should be noted that systemic administration of GLP-1 analogs, by reducing blood glucose levels, may led to similar results in terms of oxidative stress, but our study provides evidence that these effects are directly mediated by GLP-1 and cannot be attributed to an improvement of blood glucose levels. In this regard, it should be emphasized that the topical (eye drops) administration of GLP-1 does not alter the blood glucose levels and that, therefore, the effects cannot be attributed to an improvement in these levels.

In the present study, we evaluated the protein levels in the retina of some antioxidant enzymes such as glutathione peroxidase, glutathione reductase, CuZnSOD and MnSOD after topical treatment with GLP-1. We found that glutathione peroxidase and glutathione reductase were higher in diabetic mice treated with GLP-1 eye drops, but without reach the statistical significance. This result agree with those obtained by Fernández-Millán et al. who found that GLP-1 was able to enhance the activity of both enzymes in beta cells (rat INS-1E cells) [17]. Regarding CuZnSOD and MnSOD, significantly higher levels in db/db mice treated with GLP-1 eye drops in comparison with vehicle were observed. Therefore, the topical administration of GLP-1 was able to prevent the diabetes-induced downregulation of CuZnSOD and MnSOD in the retina. Overall, our findings point to the enhancement of all these antioxidant enzymes as a significant mechanism of action of GLP-1.

In order to investigate the potential role of GLP-1 in the process of DNA repair we measured the Babam2 protein, which is encoded by the *Babam2* gene, also named *Bre.* The *Babam2* gene forms the Brca1-a complex in the nucleus of multiple cell types where its function consists in repairing DNA double strands breaks. Babam2 enables the Brca1-a complex to reach DNA damage sites. Shi et al. demonstrated that the deletion of Babam2 in fibroblasts leads to the accumulation of unrepaired DNA damage [11]. In the present study, we provide first evidence that GLP-1 increases the Babam2 protein, thus suggesting that DNA repair is another pleiotropic action of GLP-1. Co-labeling with the specific neuronal marker NeuN points to the ganglion cells as the main neuronal source of the Babam2 protein. In addition, we have confirmed our previous observation that GLP-1 upregulates Ki67 (an excellent marker of cellular proliferation), which also colocalized with NeuN. Taken together these findings suggest that the antioxidant properties of GLP-1 are linked to its capacity to promote neurogenesis.

## 5. Conclusions

Topical administration of GLP-1 in an experimental model of DR (db/db mice) confers protection against the damage caused by nitro-oxidative stress. The GLP-1-mediated increase of some antioxidant enzymes such as MnSOD and CuZnSOD is an important contributing factor. The prevention of DNA/RNA damage, the increase in DNA repair and the enhancement of cellular proliferation observed after treating with GLP-1 all reinforce the concept that GLP-1 promotes neurogenesis in the diabetic retina. However, further studies not only to confirm this important issue, but also to unravel the underlying mechanisms linking the antioxidant effects and cellular proliferation are needed.

## Figures and Tables

**Table 1:** Primary antibodies, targets, specific dilutions and sources used in western blot analysis.

Antibodies	Description
Babam2	Rabbit monoclonal; 1:1000; ab177960; Abcam, Cambridge, UK
Cyclophilin A	Rabbit polyclonal; 1:10,000; BML-SA296; Enzo Life Sciences, Lausen, Switzerland
CuZnSOD	Rabbit polyclonal; 1:1000; GTX100554; GeneTex, Hsinchu, Taiwan
Gadph	Mouse monoclonal; 1:10,000; sc-32233; Santa Cruz, Dallas, Texas, USA
Glutathione peroxidase	Rabbit polyclonal; 1:1000; GTX116040; GeneTex, Hsinchu, Taiwan
Glutathione reductase	Rabbit polyclonal; 1:1000; GTX114199; GeneTex, Hsinchu, Taiwan
MnSOD	Rabbit polyclonal; 1:1000; ab13533; Abcam, Cambridge, UK
Vinculin	Mouse monoclonal; 1:7000; sc-73614; Santa Cruz, Dallas, Texas, USA

**Table 2:** Targets, dilutions and sources of applied antibodies used in the immunofluorescence analysis.

Primary Antibodies	Description
DNA/RNA damage (8-hydroxy-guanosine)	Mouse monoclonal; 1:100; ab62623; Abcam, Cambridge, UK
Babam2	Rabbit monoclonal; 1:100; ab177960; Abcam, Cambridge, UK
CuZnSOD	Rabbit polyclonal; 1:100; GTX100554; GeneTex, Hsinchu, Taiwan
Ki67	Rabbit polyclonal; 1:500; ab15580 (Abcam, Cambridge, UK)
MnSOD	Rabbit polyclonal; 1:100; ab13533; Abcam, Cambridge, UK
NeuN	Mouse monoclonal; 1:200; ab104224; Abcam, Cambridge, UK
Nitro tyrosine	Mouse monoclonal; 1:100; ab7048; Abcam, Cambridge, UK
**Secondary Antibodies**	**Description**
Alexa Fluor 488 Goat anti-mouse	Goat polyclonal; 1:600; #A-11032; Abcam, Cambridge, UK
Alexa Fluor 488 Goat anti-rabbit	Goat polyclonal; 1:600; ab150081; Abcam, Cambridge, UK
Alexa Fluor 594 Goat anti-mouse	Goat polyclonal; 1:600; ab150113; Life Technologies (Thermo Fisher Scientific) Waltham, MA, USA
Alexa Fluor 594 Goat anti-rabbit	Goat polyclonal; 1:600; A-110012; Life Technologies (Thermo Fisher Scientific) Waltham, MA, USA
